# Dissecting the determinants of depressive disorders outcome: an in depth analysis of two clinical cases

**DOI:** 10.1186/1744-859X-6-5

**Published:** 2007-02-07

**Authors:** Alessandro Serretti, Raffaella Calati, Osmano Oasi, Diana De Ronchi, Cristina Colombo

**Affiliations:** 1 Institute of Psychiatry, University of Bologna, Italy; 2 Department of Psychology, Catholic University, Milan, Italy; 3 Department of Psychiatry, San Raffaele Scientific Institute, Milan, Italy

## Abstract

Clinicians face everyday the complexity of depression. Available pharmacotherapies and psychotherapies improve patients suffering in a large part of subjects, however up to half of patients do not respond to treatment. Clinicians may forecast to a good extent if a given patient will respond or not, based on a number of data and sensations that emerge from face to face assessment. Conversely, clinical predictors of non response emerging from literature are largely unsatisfactory.

Here we try to fill this gap, suggesting a comprehensive assessment of patients that may overcome the limitation of standardized assessments and detecting the factors that plausibly contribute to so marked differences in depressive disorders outcome.

For this aim we present and discuss two clinical cases. Mr. A was an industrial manager who came to psychiatric evaluation with a severe depressive episode. His employment was demanding and the depressive episode undermined his capacity to manage it. Based on standardized assessment, Mr. A condition appeared severe and potentially dramatic. Mrs. B was a housewife who came to psychiatric evaluation with a moderate depressive episode. Literature predictors would suggest Mrs. B state as associated with a more favourable outcome.

However the clinician impression was not converging with the standardized assessment and in fact the outcome will reverse the prediction based on the initial formal standard evaluation.

Although the present report is based on two clinical cases and no generalizability is possible, a more detailed analysis of personality, temperament, defense mechanisms, self esteem, intelligence and social adjustment may allow to formalize the clinical impressions used by clinicians for biologic and pharmacologic studies.

## Background

Treatment evaluation and guidelines relies mainly on published clinical trials. Unfortunately clinicians face an everyday clinical practice that can differ in terms of efficacy and prediction of outcome. This leaded to criticize the clinical trial method [1,2]. The difference is mainly due to the fact that in the clinical practice a much higher number of variables is taken into account. In fact case reports yield much more information and are closer to clinical practice [3]. This gap is particularly troublesome for biologic and genetic research where effects are subtle and wide [4,5].

As an attempt to fill this gap we are presenting two clinical cases of depressed subjects that are much similar in terms of traditional assessment but substantially differ when a more detailed analysis is applied. This could constitute a suggestion for inclusion of such detailed assessment in clinical trials and biologic analyses.

To pursue this goal we have chosen a battery of tests that explore the whole human complexity, according to the holistic approach of the biopsychosocial model of medicine, which considers patient illness like a combination of a large quantity of biological, psychological and social factors interacting with each other [6], and according to W.H.O. concept of health, like "a complete state of physical, mental and social well-being" [7].

We have therefore considered a number of features that have been suggested, at a variable degree of certainty, as associated with outcome [8-19]. We included in the analysis heredity, intrapsychic aspects (temperament and personality traits, personality disorders, defensive mechanisms, locus of control, coping styles, self esteem), cognitive features and social features. In order to measure those features, we tried to use validated and reliable instruments, when available. Informed consent has been obtained by the two subjects in compliance with the Helsinki Declaration in the context of approval of the local ethical committee for the study.

Although a follow up of a large cohort of depressed subjects investigated at baseline would be the correct strategy to investigate this issue, practical limitations do not allow such a study to be performed. The only comprehensive naturalistic follow up to date is the STAR*D study which, with a large effort and a multicentric approach, only targets resistant depression and it includes only a very limited number of predictive variables [20]. We therefore propose a very preliminary strategy of comprehensive assessment in line with the evidence of the complex pattern of determinants of depressive disorders [21-24].

The use of this wide-ranging assessment is also motivated by the fact that clinical predictors of non response emerging from literature are largely unsatisfactory [25]; so it is currently accepted that the coexistence of a broad number of factors contributes to the resistance to therapy response and in this paper we have tried to investigate this issue.

The double aim of this paper is to suggest a comprehensive assessment of mood disorders patients that may overcome the limitation of standardized assessments and to detect factors that plausibly contribute to the well known marked differences in depressive disorders outcome.

## Mr. A

Mr. A is a fifty-year-old industrial manager. Striking politeness and respectfulness characterize him – he defines himself a "medieval knight". His inclination toward cooperation contributes to the fluency of interviews.

He describes himself like a good planner and his life style reflects it: he got a degree in engineering with full marks at twenty-five years old, something that made him very proud; at twenty-six he did military service, he took the qualifying examination and he began to work in a design laboratory of a small business; at twenty-seven he got married with a woman of the same age and they gave birth to a daughter when he was thirty, an experience that he defined hard but of immeasurable joy.

To spoil these plans several depressive episodes have cropped up. At twenty-six years old, in the period of the first employment, Mr. A began to suffer depressive symptoms: persistent sadness, loss of interest in activities, psychic anxiety, weight loss (3–4 kilograms), sleeping difficulty, especially waking too early, sluggishness, lack of energy, tiredness, inappropriate guilt and loss of confidence, thinking and concentrating difficulties. Mr. A imputed this collapse to difficulties and incomprehensions in the business framework. He came to psychiatric evaluation and he was treated with clinical management and pharmacological therapy (clomipramine, dose unknown). After the therapy response and the symtomatological remission, Mr. A got married and this event, in conjunction with the experience of paternity, helped him to become settled and to pass years of composure.

From the age of forty-five years old other three depressive episodes followed, concomitant with stress in the company context. These three episodes, with similar symptomatology of the first, occurred respectively when he was forty-five, forty-seven and forty-eight years old. Each episode was treated with clinical management and the same pharmacological medication (fluvoxamine 200–300 mg and mirtazapine 15 mg), with positive response and complete symtomatological remission. The time course of Hamilton Rating Scale for   Depression (HAM-D) scores in the first (index) episode at 45 years was 23 at baseline and in the following 7 weeks was: 23, 18, 17, 16, 8, 8, 2. The present score of his depressive symptoms   assessment, carried out with the use of the HAM-D, is 2 (at the item 6, Late Insomnia, 2 = Unable to fall asleep again if he gets out of bed).

A number of stressful life events were concomitant with the occurrence of depression, besides dissatisfactions in job; Mr. A himself made a list of the "heavy events": the death of his father, several organic diseases of his wife and daughter, the country home devastated by an earthquake, the job burden of his wife.

Moreover, two years ago, Mr. A's daughter began to show marked psychopathology which will be diagnosed as Bipolar Disorder, type I. Nevertheless, in this time, Mr. A did not show other depressive signs. He referred to feel himself changed, capable to consider events with detachment, perhaps thanks both to pharmacological treatment, which is still taking, and self-discipline learned with the help of meditation and physical activity.

So, contrary to all expectations, Mr. A condition, at the beginning apparently severe, has completely recovered and, at the present time, seems to be steady.

## Mrs. B

Mrs. B, a sixty-year-old small looking frightened woman came to psychiatric evaluation after the death of her husband, at fifty-one years. From the first interview her frailty was clear. She had few hopes about her recovery.

She felt deeply depressed and anxious, with symptoms like persistent sadness, inappropriate crying, feelings of worthlessness, hopelessness, complete loss of self esteem, loss of interest in activities, agitation and psychic anxiety, appetite and weight loss, sleeping difficulty, lack of energy, tiredness, thinking difficulty, impaired concentrating and making decisions, fear of the future, difficulties in relationships and social withdrawal.

She lived in an isolated setting, incapable to do anything. Difficulties to find the right pharmacological medication became visible quite early because of the absence of any response (amitryptiline not tolerated, amisulpiride 50 mg, citalopram 60 mg, paroxetine 50 mg, clomipramine 150 mg, pindolol 20 mg, mirtazapine 60 mg, trazodone 100 mg, lithium 600 mg, venlafaxine 375 mg, olanzapine 10 mg, fluoxetine 60 mg, all for extended periods and in various combinations).

In truth, the first distress sign came into sight when, at the age of twenty-seven, Mrs. B had an abortion. This awful experience damages her everyday-life and forced her in bed for a long time. Unfortunately, other two subsequent abortions, at twenty-nine and thirty-two years old, shocked Mrs. B. She described this period like characterized by ups and downs: the delighted moments during pregnancy and the deep grief of lost and mourning followed one upon the other without a break. Besides feeling depressed, Mrs. B suffered of panic symptomatology (racing heartbeat, excessive sweating, trembling, breathlessness, chest discomfort, nausea, dizziness, feeling of derealization, fear of losing control), which impaired her life, compelling her to avoid crowded places and circumstances like travel by underground, tram or air.

Providentially, at the age of thirty-four years old, Mrs. B gave birth to a son. She stopped to work (she was a tailor) and devoted herself to her son. The uneasiness feelings considerably diminished, even though anxiety and panic attacks were always present.

Nevertheless, after her husband death, her condition got worse and, at the present time, no treatment, neither clinical management nor pharmacological therapy, has any effects on mood and anxiety symptomatology. The present score of her depressive symptoms assessment, carried out with HAM-D, is 24. The score is substantially stable over time.

Besides the three abortions and the loss of her husband, the death of both parents and two brothers has contributed to Mrs. B manifestation of depression.

Also regarding Mrs. B condition, expectations based on standard research criteria, in this case of a good response, were misleading.

### Hereditary features

In accordance to the principles of formal genetics, sharing a portion of genetic heritage increases the risk of being affected by the same disease.

Both Mr. A and Mrs. B have other cases of depressive disorders in their families, but with substantial differences: Mr. A mother was affected by depressive disorder and showed an anxious temperament; moreover, the bipolar disorder of Mr. A daughter strengthen the genetic hypothesis. On the contrary, only Mrs. B mother aunt was affected by depression and anxiety, pharmacologically treated. Therefore, the genetic load is more marked in Mr. A compared to Mrs. B. This is usually an indication of more 'typical' mood disorder compared to sporadic cases [26] and it has been described as more responsive to treatments [27,28].

### Intrapsychic features

#### Temperament and personality traits

Personality can be defined as a complex of psychological and behavioural dimensions [29,30]. Several theories attempted to define what is personality and descriptions of human personality are so many as theories are. Among these, the bio-social theory of Cloninger gave an original and successful contribution, describing a model that incorporates both biological and socio-cultural influences in the development of human personality [31]. His model was based on the assumption that a part of the individual's personality is heritable. In particular, he hypothesized that personality is composed both by *Temperament*, the totality of traits which are heritable and stable throughout life, and *Character*, the whole traits that are influenced by socio-cultural learning and that mature throughout life. *Temperament *consists of four traits, so called Harm Avoidance, Novelty Seeking, Reward Dependence and Persistence. Harm Avoidance denotes the individual's inclination to behavioral inhibition in front of potentially dangerous stimuli and to anticipate negative effects; Novelty Seeking relates to exploratory behaviors and activation in response to novel stimuli; Reward Dependence concerns relational and affective skills but also other dependencies; finally Persistence characterizes industrious, hard working and stable individuals despite frustration and fatigue. *Character *consists of three dimensions: Self-Directedness, Cooperativeness and Self-Transcendence. Self-Directedness expresses the individual's competence towards autonomy, reliability and maturity; Cooperativeness is related to social skills, like support, collaboration and partnership; finally, Self-Transcendence denotes the aptitude towards mysticism, religion and idealism.

The Temperament and Character Inventory (TCI), a 240 items tool to assess individuals differences in the seven basic dimensions of *Temperament *and *Character *[32], was administered to both Mr. A and Mrs. B (Table 1). Mr. A showed high scores in Harm Avoidance (100), Reward Dependence (104), Persistence (126), Self-Directedness (146) and Cooperativeness (132) and low scores in Novelty Seeking (87) and Self-Transcendence (50). Mrs. B showed similar scores to Mr. A in Reward Dependence (109) and Novelty Seeking (86). In comparison with Mr. A, she had higher scores in Harm Avoidance (128), Cooperativeness (147) and Self-Transcendence (66), even if Self-Transcendence score remains low, and she had lower scores in Self-Directedness (138) and Persistence (103).

**Table 1:** Mr. A and Mrs. B TCI scores in comparison with minimum and maximum values.

**Temperament and Character dimensions**	**Minimum Scores**	**Mr. A**	**Mrs. B**	**Maximum Scores**
Harm Avoidance	33	100	128	165
Novelty Seeking	35	87	86	175
Reward Dependence	30	104	109	150
Persistence	35	126	103	175
Self-Directedness	40	146	138	200
Cooperativeness	36	132	147	180
Self-Transcendence	26	50	66	130

So, Mr. A appears quite inhibited and responsible, purposeful, goal-oriented and resolute. Differently, Mrs. B seems to be much more timorous and inhibited toward potentially dangerous stimuli or social circumstances, and less mature and tenacious, although more collaborative.

Numerous studies have found high scores in Harm Avoidance trait in samples of patients affected by mood disorders [32-35]; this fact fortifies the hypothesis of a link between depression and withdrawal like reaction to loss or disappointment [36].

Moreover, also low Novelty Seeking and low Self-Directedness represent trait markers for liability to recurrent major depressive disorder [34,35,37].

Therefore, we can hypothesize that the higher introversion and lower responsibility and maturity of Mrs. B could have contributed to the negative outcome of therapies. Nevertheless, it must be said that Harm Avoidance trait is gender-specific and generally scores are higher in women than men [38-42]. Moreover high Harm Avoidance scores could be directly related to the depressive symptomatology [32].

#### Personality disorders

Both Mr. A and Mrs. B were investigated for Axis II diagnoses using the Structured Clinical Interview for the DSM-IV (SCID-II) [43].

Mr. A suffers from an Obsessive-Compulsive Personality Disorder, with symptoms like: excessive attention to details, rules, lists, tidiness, organization, plans; excessive conscientiousness, meticulousness, rigorousness and idealism; incapability to get rid of consumed and no value objects; rigidity and obstinacy.

Differently, Mrs. B has an Avoidant Personality Disorder, with traits like: avoidance of job activities that imply significant interpersonal relationships due to the fear of criticism and judgment; avoidance of interpersonal relationships if there is no certainty of being accepted; inhibition in interpersonal relationships and inadequacy feelings; feelings of inferiority; reluctance toward new activities. Moreover, Mrs. B shows a number of traits of Dependent Personality Disorder (like difficulties to express disagreement, difficulty to do things autonomously, fear of being alone and need of support) and several traits of Obsessive-Compulsive Personality Disorder (perfectionism interfering with completing activities, excessive conscientiousness and idealism; incapability to get rid of consumed and no value objects).

In literature, up till now, there is evidence of the fact that the occurrence of a personality disorder is high among depressive disorders [44] and complicates their treatment [45,46], though evidence is not unequivocal [47].

In particular, Cluster C Personality Disorders, including Avoidant, Dependent and Obsessive-Compulsive subtypes, has been largely investigated. Firstly, Cluster C subtypes seem to predominate between personality disorders in mood disorder samples [48-52]. Secondly, it was observed that a Cluster C diagnosis was associated with significantly higher rates of early-onset depression [49]. Several recent studies have replicated these findings: Nubukpo and colleagues observed that the frequency of personality disorders was higher in patients with early-onset depression rather than in those with late-onset depression; moreover, between the early-onset depressed patients, the most frequent personality disorders were Avoidant and Dependent [53]. Thirdly, patients with both panic disorder and major depression showed higher Harm Avoidance levels and a greater prevalence of Cluster C personality disorders, compared to patients with pure disorders [54]. Moreover, Russell and colleagues, in a study previously mentioned, observed that a Cluster C diagnosis was associated with comorbid anxiety disorder [49].

Finally, Cluster C subtypes emerged as robust predictors of slowed remission from major depressive disorder. In two different studies Viinamaki and collaborators investigated whether Cluster C personality disorder is associated with recovery from depression and found an association between lack of recovery and presence of Cluster C personality disorder. In detail, among patients with depression alone, 54% had recovered from the disorder, but only 16% of those with a Cluster C personality disorder and depression recovered [55,56]. Grilo and colleagues observed that participants with major depressive disorder who had certain forms of coexisting personality disorder psychopathology (Avoidant, Schizotypal or Borderline) had a significantly longer time to remission from depression than did patients without any personality disorder [57]. Moreover, Morse and colleagues observed that Cluster C was associated with longer time-to-response during acute treatment and non-response in continuation or maintenance treatment. Although not statistically significant, there was evidence of a cumulative negative impact of Cluster C personality disorder and residual depressive symptoms on instrumental activities of daily living during maintenance treatment [58].

Also negative results were reported: in a sample of depressed patients, one comorbid personality disorder was of limited relevance to the course of the affective illness, especially if it was a Cluster C personality disorder [59].

Nevertheless, summarizing, the large quantity of positive studies justifies the assumption that the diagnosis of a Cluster C personality disorder could be associated with early-onset depression and comorbid anxiety disorder and it hinders the alleviation of depressive symptoms in major depression.

Consequently, we can hypothesize that Mrs. B repeated treatment failures was due to the specific structure of her personality, in which coincident traits of three personality disorders have been crystallized in a maladaptive organization. These conclusions could be connected to temperamental considerations: actually, Cluster C personality disorders were found related just with high Harm Avoidance, low Novelty Seeking and low Self-Directedness [60], therefore this fact makes Mrs. B personality profile emblematic.

For what concerns Mr. A, his personality organization appears more adaptive: in fact, he shows only one personality Disorder – Obsessive-Compulsive – which furthermore probably represents an important resource for him, especially in the job field.

#### Defense mechanisms

We have also considered the defense mechanisms of Mr. A and Mrs. B, administering them the 88 items Defense Style Questionnaire (DSQ) by M. Bond [61], recently validated on Italian sample [62]. The questionnaire allows the identification of four defensive mechanism styles, representing groups of defenses classified from more immature, and therefore maladaptive, to more mature and adaptive (Table 2).

**Table 2:** The defensive styles according to Bond [61].

**Style 1**: Reflects a regressive situation and highlights behavioural disorders. The patient appears incapable of integrating his own impulses in a constructive and responsible action. It includes defenses that are commonly considered immature
**Autistic withdrawal, acting-out, inhibition, passive aggression, projection**

**Style 2**: Identifies problems in relationships and includes defenses that "distort the image" more than defenses concerning action. Such a defensive structure disturbs the object relations while it does not interfere with social and work fulfilment; in literature these are defenses associated with borderline and narcissistic disorders

**Splitting, primitive idealization, omnipotent devaluation**

**Style 3**: Includes "self-sacrificing" defenses (for instance the compulsion to "appear good"); it poses problems more on the level of creative capabilities rather than relational ones, allowing in this last field stable object relations even if not necessarily "healthy" ones (i.e. masochistic relations)

**Reactive formation, pseudo-altruism**

**Style 4**: It is also defined as "adaptive"; including defenses associated with a good adjustment and a good integration

**Sense of humour, repression, sublimation**

This questionnaire has consented us to analyze the prevalent defensive styles of Mr. A and Mrs. B (Table 3). Their scores are similar to those of healthy Italian sample [62], with the exceptions of Mr. A scores in Anticipation and Sublimation and Mrs. B scores in Reactive Formation, Inhibition and Isolation, higher in comparison with those of healthy sample.

**Table 3:** Mr. A and Mrs. B DSQ mean scores and healthy sample mean scores. The asterisk indicates deviance from normal values on the basis of standardized distance from the population mean and significance of the mechanism on the basis of the number of items.

**Defense Mechanisms**	**Healthy Men Sample Scores (Mean ± SD)**	**Mr. A Scores**	**Healthy Women Sample Scores (Mean ± SD)**	**Mrs. B Scores**
Acting-out	3.52 ± 1.77	4.8	4.06 ± 1.66	4.4
Affiliation	2.79 ± 2.05	5	3.48 ± 2.24	5
Undoing	2.67 ± 1.67	2	2.60 ± 1.80	4.33
Anticipation	4.86 ± 2.10	7.5*	4.97 ± 2.13	6.5
Passive aggressive	2.74 ± 1.47	2.8	2.81 ± 1.45	2.4
Consumption	1.94 ± 1.67	2.33	2.56 ± 1.70	1.33
Denial	1.80 ± 1.29	3.5	1.43 ± 1.32	3
Fantasy	4.52 ± 2.87	6	4.78 ± 2.83	5
Reaction formation	2.80 ± 1.60	3.8	2.93 ± 1.60	5.2*
Primitive idealization	3.14 ± 2.29	4	3.62 ± 2.58	6.5
Projective identification	0.98 ± 1.86	1	1.51 ± 2.45	5
Inhibition	2.96 ± 1.72	3.8	3.56 ± 1.84	7*
Isolation	3.10 ± 1.65	3	2.47 ± 1.59	4.5*
Help-rejecting complaining	2.22 ± 1.97	2	2.28 ± 1.96	4
Omnipotence	2.71 ± 1.61	2.5	2.27 ± 1.58	1.33
Task-orientation	4.87 ± 2.37	6.5	5.18 ± 2.12	2.5
Projection	1.62 ± 1.06	2.44	1.79 ± 1.19	2.44
Pseudo-altruism	5.69 ± 2.10	7	6.22 ± 1.91	8
Regression	2.30 ± 2.02	5	3.31 ± 2.12	6.5
Suppression	3.94 ± 2.08	5	3.58 ± 2.18	3
Withdrawal	4.56 ± 2.05	6.33	5.47 ± 1.93	7.33
Splitting	3.45 ± 2.09	4	3.40 ± 1.98	4.67
Somatization	1.97 ± 2.09	4	2.97 ± 2.28	5.5
Sublimation	2.05 ± 2.65	5*	2.60 ± 2.98	5
Humor	4.69 ± 1.84	4.33	4.57 ± 1.93	4.33

Analysing scores different from the control sample, two Mr. A defensive mechanisms are more adaptive. Anticipation and Sublimation, in which he obtained higher scores, are mature defenses. Mr. A usually faces up to emotional conflicts or internal and external stressful life events in two adaptive way: 1) anticipating and prefiguring his affective reactions towards future possible events or anticipating the consequences and the solutions of these events (Anticipation); 2) channeling potentially maladaptive affects and impulses in socially appreciated behaviors, like sport, sculpture and painting (Sublimation). Abraham was the first who underlined the possible link between depression and specific defenses like sublimation: he describes in a brilliant way how the painter Giovanni Segantini recreated in his works the love for his mother [63].

On the contrary, several Mrs. B defensive mechanisms appear maladaptive. Reactive Formation, Inhibition and Isolation are neurotic immature defenses. Mrs. B usually faces up emotional conflicts or internal and external stressful life events in three maladaptive way: 1) with behaviours, thoughts and affects opposite to her own unacceptable thoughts and feelings (Reactive Formation); 2) reducing relational capacity to avoid the anxiety associated to unacceptable internal conflicts (Inhibition); 3) removing affects related to concepts and maintaining only cognitive elements (Isolation). M. Klein, in her first studies about early anxieties, placed two different defense mechanisms like Isolation and Splitting close together: it can suggest that the psychological condition of Mrs. B is nearer to a higher level of loss anxiety and it needs early defenses [64].

We can hypothesize that the maturity of Mr. A defenses has a protective function, while the immaturity of Mrs. B defenses could be a further factor explaining the absence of any therapy response. In fact, in the same line of evidence, Mullen and collaborators, comparing treatment responders and non-responders of a major depressive disorder sample, found that medication responders used significantly less maladaptive defenses than did non-responders and had a significantly higher or healthier level of overall defensive functioning [65]. Nevertheless, it is essential to underline that the individual defensive style could be also modulated by depressive mood itself. Moreover, in a study over mentioned, immature defenses seemed to be strongly related to low Self-Directedness and both Self-Directedness scores and immature defense scores were predictive of the presence and number of personality disorders [60]. Mrs. B particular profile supports these data.

#### Locus of control

We have also considered the locus of control of Mr. A and Mrs. B, administering them the 24 item Internal, Powerful Others and Chance Scales (IPC Scales) by H. Levenson [66]. The scale has been validated on Italian sample [67].

Locus of control refers to an individual's generalized expectations concerning where control over subsequent events resides. Hannah Levenson offered an alternative model of Rotter's original locus of control formulation [68]. Whereas Rotter's conceptualization viewed locus of control as unidimensional (internal to external), Levenson's model asserts that there are three independent dimensions: Internal, Powerful Others and Chance. According to Levenson's model, one can endorse each of these dimensions of locus of control independently and at the same time. For example, a person might simultaneously believe that both oneself and powerful others influence outcomes, but that chance does not. The IPC Scales allow the identification of the three locus of control dimensions.

Mr. A and Mrs. B scores are similar to those of healthy Italian sample [67] (Table 4).

**Table 4:** Mr. A and Mrs. B IPC Scales mean scores and healthy sample mean scores.

**Locus of Control scales**	**Healthy Men Sample Scores (Mean ± SD)**	**Mr. A Scores**	**Healthy Women Sample Scores (Mean ± SD)**	**Mrs. B Scores**
Internal	32.54 ± 8.35	40	30.35 ± 9.12	28
Powerful Others	18.16 ± 8.59	8	17.04 ± 8.67	9
Chance	19.16 ± 8.92	18	20.94 ± 8.40	25

Nevertheless, Mr. A Internal score is higher than Mrs. B one (40 versus 28) and Mr. A Chance score is lower (18 versus 25). The prominent internal locus of control of Mr. A represents a resource: he is certain to control events of his own life, to obtain success thanks to hard work and to his own capacities and talent. Mrs. B has a less strong internal locus of control and she scarcely believes to the influence of fortune in determining her life.

It is essential to consider that these features could also be altered by the specific disorder outcome: Mr. A positive response and complete stable recover could have contributed to his confidence, while Mrs. B repeated unsuccessful treatments have certainly emphasized her feelings of powerlessness.

#### Coping styles

Besides, we have considered the coping styles of Mr. A and Mrs. B, administering them the 28 items Brief COPE by Carver [69] (Table 5). It has not been validated in Italy. The questionnaire allows the identification of fourteen coping styles: Positive Reorganization, Attention Withdraw, Expression, Instrumental Support, Operatively Facing Up, Negation, Religion, Humor, Behavioral Disengagement, Emotional Support, Substance Use, Acceptation, Planning, Self Blaming.

**Table 5:** Mr. A and Mrs. B Brief COPE mean scores. The asterisk indicates marked differences between the two patients (≥ 4).

**Coping Styles**	**Mr. A Scores**	**Mrs. B Scores**
Positive Reorganization	4	6
Attention Withdraw	2	5
Expression	7	5
Instrumental Support	2	7*
Operatively Facing Up	8*	4
Negation	2	3
Religion	2	8*
Humor	4	5
Behavioral Disengagement	2	5
Emotional Support	6	5
Substance Use	2	2
Acceptation	8*	4
Planning	8*	4
Self Blaming	7	4

We focused our attention on marked differences between the two patients (≥ 4). Mr. A uses more adaptive and pragmatic coping strategies like Operatively Facing Up, Acceptation and Planning. Nevertheless, Mrs. B seems to have a positive, essential resource too: the support of Religion. Moreover, she usually looks for advices and aids from others (Instrumental Support); this coping style could be the result of Mrs. B dependent personality traits (like difficulties to do things autonomously).

#### Self esteem

To assess Mr. A and Mrs. B self esteem we have administered them the 10 items Self Esteem Scale by Rosenberg [70]. We would expect to observe Mrs. B scores lower than Mr. A ones, also considering her depressive symptomatology. Nonetheless, contrary to all expectations, their self esteem level did not differ. This fact is contrasting with the observation of lower self esteem in euthymic depressed subjects [71] and we are unable to explain this other that some contingent factor that could have influenced it.

### Cognitive features

The Wechsler Adult Intelligence Scale – Revised (WAIS-R) [72] was administered to Mr. A and Mrs. B to evaluate their cognitive functioning and their intelligence quotient.

Mr. A Total IQ was 136, Verbal IQ 126 and Performance IQ 138; Mrs. B obtained lower scores: Total IQ was 112, Verbal IQ 104 and Performance IQ 121. Mr. A scores would suggest that he has more cognitive resources than Mrs. B, but, considering that WAIS-R assesses also the individuals education level, we could observe that the differences between the two scores could be due to the disparity of Mr. A and Mrs. B education years (18 in the case of Mr. A versus 5 in the case of Mrs. B). Furthermore, their different occupations, in terms of cognitive involvement, (industrial manager versus housewife) could influence the outcome.

Finally, cognitive function has been found impaired during acute episodes, particularly attention, learning and memory, psychomotor functioning and frontal executive functions [73] and this could be another possible explanation of the difference in the two scores [74]. Considering all these observations, it is possible to state that both patients have good cognitive resources.

### Social features

#### Social adjustment

We have also considered the social adjustment of Mr. A and Mrs. B (Table 6), administering them the Social Adjustment Scale Self-Report (SAS-SR) [75]. The questionnaire has been validated in many countries including Italy and it evaluates six adjustment areas: Work, Spare Time, Family, Children, Family Unity, Finance.

**Table 6:** Mr. A and Mrs. B SAS-SR mean scores.

**Social Adjustment Areas**	**Healthy Sample Scores (Mean ± SD)**	**Mr. A Scores**	**Mrs. B Scores**
Work	1.24 ± 0.56	1.5	1.7
Spare Time	1.77 ± 0.43	1.8	3.2
Family	1.56 ± 0.39	1.2	1.4
Children	0.76 ± 0.80	1	1.5
Family Unity	1.07 ± 0.68	1	1.7
Finance	1.25 ± 0.56	1	2

Considering the fact that higher scores correspond to higher impairment, we can observe that Mrs. B reported scores that evidence some impairment in the social functioning. This has been previously observed for patients with mood disorder even in their remission phase [71,76]. Mr. B functioning, compared with control one, is worse in all areas, with the exception of Family field. Moreover, comparing Mr. A and Mrs. B scores, a relevant divergence could be detected in the Spare Time area (1.8 versus 3.2).

Since social functioning can be evaluated as an outcome of treatment [77], Mrs. B higher social impairment has surely been modulated by the absence of any positive effect.

#### Morningness-eveningness preference

Finally, we have evaluated Mr. A and Mrs. B morningness or eveningness preference administering them the Morningness-Eveningness Self-Assessment Questionnaire [78].

Since mood disorders are characterized by circadian rhythm abnormalities [79], we tried to analyze both Mr. A and Mrs. B rhythm profile. Mr. A reported morningness preference scores markedly higher than Mrs. B one (71 versus 47).

Recent studies showed that a single nucleotide polymorphism (T3111C), located in the 3' flanking region of the human CLOCK gene, was associated with diurnal preferences of human healthy subjects, with higher eveningness in subjects carrying at least one copy of the C allele [80]. In another study the possible role of the same polymorphism in the regulation of diurnal mood fluctuations during a major depressive episode was investigated; Authors observed a significantly worse outcome in homozygotes for the C variant [81].

Consequently, it is possible to hypothesize a link between eveningness and higher recurrence and, also in this case, Mrs. B condition could be representative of this connection.

## Conclusion

This manuscript aimed to analyse in depth the mixture of aspects contributing to depressive disorders outcome. It is interesting to consider that, from the standard assessment point of view, Mr. A and Mrs. B differ one from another only for what concerns therapy response: both are affected by recurrent major depression, no major somatic or neurologic disorder is present, no other DSM-IV axis I comorbidity. Subsequently, patients with so divergent clinical history in standard research terms are similar. On the contrary, the complexity and heterogeneity of the individual case should be meticulously taken into account.

Summarizing, we can consider Mr. A depression like adaptive since it has facilitated detachment and a more balanced involvement in his life. In fact, depressive disorder has long been explored in terms of adaptive and maladaptive functions [82]. Some depressive disorders, at mild levels, can be adaptive if they enable individuals to disengage from aversive environments and to relocate or elicit new resources from the environment [83-85]. Moreover, Mr. A meticulousness and his strict involvement in working area could have an essential protective function for him.

On the contrary, in Mrs. B case depression has maladaptive functions. The impact of prior pharmacological interventions on Mrs. B may have been adversely affected by several factors: 1) personality factors such as high Harm Avoidance and low Novelty Seeking and Self-Directedness; 2) Avoidant Personality Disorder, which prevents Mrs. B from putting her energy in new social situations; 3) Dependent Personality traits and their combination with the loss of her husband; 4) immature defensive mechanisms at intrapsychic level; 5) a therapeutic alliance probably based on omnipotence attributions. We can also hypothesize a different way to react to previous losses and aversive environments: Abraham indicates, among the factors of melancholia, the repeating of situations of loss and mourning [86]. This different way can be found in specific personality organization in which is very difficult to promote the change [87].

Subsequently, we could notice that the role of intrapsychic factors as clinical predictors of non response appears fundamental in the cases presented, especially for what concerns the constellation of individual temperament and personality traits, personality disorders, defensive mechanisms and locus of control. Nevertheless, this presentation has only a suggestive aim, given that no formal (statistical) demonstration has been provided of the predictive value of the reported factors. The differences we observed could be due to chance variations, however we observed associations with poor outcome that were in the direction hypothesized by the a-priori knowledge (e.g. dependent personality profile, lack of maturity, lack of social support) but that have never been joined in a comprehensive assessment.

This last point is the main limitation of our paper: as we stated in the introduction section we did not perform a large, prospective, cohort study with a comprehensive assessment. Such a study would require an extraordinary organizational and economic effort. Even the largest funding agency available to date did only organize a much smaller follow up [20]. We are also aware that two subjects, of different sex, can be only described and no generalizability is possible.

The choice of the test is also a crucial point. A number of features could be measured with a number of instruments. This article is not aimed for a review of all possible predictors [10,13,14,16,17]. We followed the guideline of investigating features previously associated with outcome and using validated instruments used in previous studies. The indications we reported may therefore be of use for larger studies where some of the features we propose could be included. This would improve informativeness and generalizability of clinical trial results [1,88].

Further, a more detailed dissection of depressive status could be of benefit for biologic and specifically genetic studies, where the small variances explained by single gene variant require a careful control of environmental confounders [4]. Alternatively genes may themselves control for basic features [89] such as temperament [90,91], drug response [92], IQ [93], or complex combinations of features [5].

In conclusion, we suggest that the inclusion of a set of assessment that more deeply investigate the patient status may help in filling the gap between routine clinical activity and standardized assessments for pharmacologic or biologic studies.

## Key points

- Clinical trial samples are scarcely representative of 'real' patients

- Standardized clinical assessment is very limited and does not take into account many subtle variables that predict antidepressant response in the everyday clinical practice

- Those variables include personality, temperament, defense mechanisms, self esteem and social adjustment

- Inclusion of those variables in the evaluation is costly but increases validity and representativity for clinical and biologic studies

## Competing interests

The author(s) declare that they have no competing interests.

## Authors' contributions

AS conceived of the study, and participated in its design and coordination and helped to draft the manuscript. RC drafted the manuscript. OO drafted and supervised the psychoanalytic sections. DD drafted the personality sections. CC drafted conclusions and supervised the clinical process. All authors read and approved the final manuscript.
